# Association of lncRNA PVT1 Gene Polymorphisms with the Risk of Essential Hypertension in Chinese Population

**DOI:** 10.1155/2022/9976909

**Published:** 2022-01-06

**Authors:** Rong Li, Xia Yu, Yang Chen, Mulun Xiao, Meiling Zuo, Yuanlin Xie, Zhousheng Yang, Dabin Kuang

**Affiliations:** ^1^Institute of Pharmacy & Pharmacology and the Second Affiliated Hospital, University of South China, Hengyang 421001, China; ^2^Department of Urology, The First Affiliated Hospital of Zhengzhou University, Zhengzhou 450000, China; ^3^The Affiliated Changsha Hospital of Hunan Normal University, Changsha, Hunan 410006, China; ^4^Department of Pharmacy, The People's Hospital of Guangxi Zhuang Autonomous Region, Nanning, China

## Abstract

Vascular dysfunction and hyperlipidemia are essential risk factors contributing to essential hypertension (EH). The plasmacytoma variant translocation 1 (PVT1) is involved in modulating angiogenesis in tumor tissues and plays an important role in fat differentiation in the progress of obesity. Therefore, we selected two tagSNPs of PVT1 (rs10956390 and rs80177647) to investigate whether they are contributing to the risk of hypertension in Chinese patients. In total, 524 adult patients with EH and 439 matched healthy controls were enrolled for two central of China. *Results*. PVT1 rs10956390 and rs80177647 polymorphisms were genotyped by using TaqMan assay. PVT1 rs10956390 TT genotype was associated with a decreased risk of EH (OR = 0.561, 95% CI = 0.372-0.846, *P* = 0.006), while rs80177647 TA genotype was associated with an increased risk (OR = 2.236, 95% CI = 1.515-3.301, *P* < 0.001). Rs10956390 T allele was associated with lower triglyceride levels in the plasma both from healthy and EH donors. What is more, there is an association between rs10956390 polymorphism and HDL-C level, as well as LDL-C. *Conclusion*. PVT1 rs10956390 and rs80177647 polymorphisms may contribute to the risk of EH in Chinese population by regulating blood lipid levels.

## 1. Introduction

Essential hypertension (EH), a noninfectious multifactorial disease, is a crucial risk factor for the morbidity and mortality of cardiovascular disease worldwide [1]. For the low treatment rate, control rate, and poor prognosis in EH patients, there is an urgent need to reveal the complex mechanism of EH. Over decades, accumulating evidence has confirmed that the factors causing hypertension include gene-gene and gene-environment interactions as well as biological systems [2].

Long noncoding RNAs (lncRNAs) are a class of noncoding RNAs longer than 200 nt. After long-time defined as “gene desert” for a long time, it has been recognized that lncRNAs play a pivotal role in diseases including cardiovascular diseases in a delicate and sophisticated network modulation manner [3–6]. The plasmacytoma variant translocation 1 (PVT1) is located at cancer risk region 8q24.21 [7], which has been identified to aberrantly expressed in various cancers including pancreatic [8], prostate [9, 10], bladder [11] cancers, and hepatocellular carcinoma [12]. Zhao et al. found that PVT1 activates STAT3/VEGFA signaling axis to boost angiogenesis in gastric cancer [13]. Similarly, Zheng et al. revealed that PVT1 orchestrates the angiogenesis of vascular endothelial cells by evoking connective tissue growth factor (CTGF) and angiopoietin 2 (ANGPT2) expression in a miR-26b dependent manner [14]. Sun et al. found that PVT1 reduces the expression of miR-190a-5p in vascular endothelial cells (ECs), resulting in proliferation [15]. Guo et al. unraveled that PVT1 knockdown ameliorates ox-LDL-induced vascular endothelial cell injury and atherosclerosis through the miR-153-3p/GRB2 axis [16]. Furthermore, recent studies have shown that PVT1 was found to be a potential biomarker for obesity treatment [17]. The potential mechanism in the interaction between obesity, atherosclerosis, and hypertension is elaborated and sophisticated, which contains activation of the sympathetic nervous system, epithelial dysfunction and oxidative stress, leptin, and adiponectin [18]. In the light of all the above, we assume that PVT1 may regulate biological processes of angiogenesis abnormality-associated diseases including diabetes, obesity, and hypertension. However, it is not clear whether PVT1 can regulate hypertension by affecting the blood lipid levels in the human plasma.

Accumulating evidence has shown that genetic polymorphism may be a novel treatment strategy to improve the control and management of diseases. Notably, in a recent study, Yan et al. revealed that PVT1 rs4410871 was a protective factor for coronary heart disease (CHD) susceptibility in Chinese population and influenced the complications (hypertension or diabetes) [19]. Considering the key role of PVT1 in affecting angiogenesis and regulating obesity, PVT1 may be a potential candidate gene related to EH risk. However, there is no report on the correlation between PVT1 polymorphism and EH risk. To clarify the clinical relevance of PVT1 polymorphisms, we conducted a case-control study in this article to investigate the association between PVT1 polymorphisms and the risk of EH in Chinese population. Meanwhile, we are trying to provide a promising biomarker in the subsequent occurrence mechanism of EH and improve the prognosis for patients.

## 2. Materials and Methods

### 2.1. Subjects

261 EH patients and 294 healthy controls were consecutively recruited between January 2021 and March 2021 from the second affiliated hospital of south China. 263 EH patients and 145 control individuals were enrolled between January 2018 and December 2019 from the First People's Hospital of Jining City, Shandong Province, in the north of China. The information of all participants including gender, age, BMI, smoking history, blood pressure, triglyceride (TG), total cholesterol (TC), high-density lipoprotein (HDL), low-density lipoprotein (LDL), and blood glucose was obtained. The presence of hypertension was clinically defined as having a systolic blood pressure (SBP) of at least 140 mmHg and a diastolic blood pressure (DBP) of at least 90 mmHg (without any antihypertensive medication), 30 years ≤ age ≤ 70 years, and the course of hypertension between 1 year and 15 years. The exclusion criteria were as follows: severe organic lesions, with other malignancies, secondary hypertension, and recent history of glucocorticoid use. The healthy subjects undergoing routine healthy examinations were enrolled in the same period. This study protocol was approved by the Medical Ethics Committee of hospital involved in the study, and the written informed consent was gained from all participants or their first-degree relatives.

### 2.2. Genomic DNA Extraction and Genotyping

EDTA anticoagulation tubes were used to collect the peripheral blood samples of the subjects and stored at -20°C until analysis. Use the E.Z.N.A.TM Blood DNA Midi kit (D3494, Omega) to extract and purify the genomic DNA following the standard protocol of the kit.

Genetic polymorphisms were screened by the 1000 Genomes Project (http://www.internationalgenome.org/). Haploview 4.2 was used to select according to CHB and CHS, and the minimum allele frequency (Minor Allele Frequency (MAF)) was greater than 5%. At last, we selected two PVT1 tagSNPs (rs10956390 and rs80177647).

Genomic DNA was diluted to working concentrations of 20 ng/L for genotyping. The assay was performed utilizing TaqMan™ Genotyping Master Mix (4371355, Thermo) as recommended by the manufacturer. Assay ID for rs10956390 is C__317048_10 (Thermo), and assay ID for rs80177647 is C__101092625_10 (Thermo). SNP genotyping was performed by TaqMan real-time PCR system as reported elsewhere. And the reaction was conducted under the following conditions: 95°C for 10 min, followed by 45 two-step cycles of 95°C for 15 s and 60°C for 1 min.

### 2.3. Statistical Assay

Statistical analysis was performed using SPSS 19.0 (version 19.0 for Windows; Chicago, IL, USA). Continuous variables were presented as mean ± standard deviation (SD) or the mean ± standard error of the mean (SEM). Student *t*-tests were conducted to analyze the differences between two groups, while the significance of differences among multiple groups was evaluated using ANOVA. Hardy-Weinberg Equilibrium (HWE) was done by using *χ*^2^ test to validate the genotype frequency. Logistic regression was used to determine the association between PVT1 polymorphism and the risk of EH adjusting for multiple EH risk factors, such as the smoking history and blood glucose. In genetic association analysis, we used additive, dominant, and recessive genetic models. A two-tailed *P* value < 0.05 was considered as statistically significant.

## 3. Results

### 3.1. The Baseline Characteristics of the Subjects

The demographic and clinical characteristics of EH and control subjects were shown in Table 1. A total of 963 subjects (535 males, 428 females), with a mean age of 53 ± 8 years old, were selected including 524 EH patients and 439 control subjects. In cases, there are 261 and 263 EH patients from the south and north of China, respectively. The distribution of smoking history and the mean of body mass index (BMI, *P* < 0.001), fasting blood glucose (FBG, *P* < 0.001), serum low-density lipoprotein cholesterol (LDL-C, *P* < 0.001), systolic blood pressure (SBP, *P* < 0.001), diastolic blood pressure (DBP, *P* < 0.001), and total cholesterol (TC, *P* < 0.001) were also significantly different between cases and controls. However, triglyceride (TG, *P* = 0.412) is no significant association between cases and controls in the north of China, and high-density lipoprotein cholesterol (HDL-C, *P* = 0.714) is no significant association between cases and controls in the south of China.

Genotype distribution of the PVT1 rs10956390 and rs80177647 polymorphisms in both controls and EH patients were in agreement with the Hardy-Weinberg Equilibrium (Table 2).

### 3.2. Association of PVT1 rs10956390 C>T and rs80177647 T>A Polymorphisms and Risk for EH in the South of China

Table 3 shows the association between PVT1 polymorphisms and EH risk in the south of China. Logistic regression analysis showed that rs10956390 CT and TT genotypes were associated with decreased risk of EH (additive model: CT: odds ratio (OR) = 0.657, 95% confidence interval (CI) = 0.445-0.968, *P* = 0.034; TT: OR = 0.571, 95% CI = 0.355-0.918, *P* = 0.021; dominant model: OR = 0.629, 95% CI = 0.437-0.906, *P* = 0.013). However, no significant association was observed after adjustment for age, gender, BMI, smoking history, FBG, and dyslipidemia.

In addition, we also found that rs80177647 A allele was associated with increased risk of EH (additive model: TA: OR = 1.855, 95% CI = 1.181-2.914, *P* = 0.007; recessive model: OR = 1.842, 95% CI = 1.188-2.855, *P* = 0.006). Moreover, the adjusted result was also significant (additive model: TA: OR = 1.950, 95% CI = 1.023-3.781, *P* = 0.042; dominant model: OR = 1.956, 95% CI = 1.041-3.676, *P* = 0.037).

### 3.3. Association of PVT1 rs10956390 C>T and rs80177647 T>A Polymorphisms and Risk for EH in the North of China

Logistic regression analyses revealed that EH risk was decreased significantly in carriers of T allele of rs10956390 polymorphism than CC genotype (additive model: CT: OR = 0.525, 95% CI = 0.321-0.860, *P* = 0.010; TT: OR = 0.402, 95% CI = 0.228-0.712, *P* = 0.002; dominant model: OR = 0.480, 95% CI = 0.303-0.762, *P* = 0.002; recessive model: OR = 0.595, 95% CI = 0.370-0.957, *P* = 0.032) as shown in Table 4. And after adjustment, the association was still significant (additive model: CT: OR = 0.409, 95% CI = 0.221-0.757, *P* = 0.004; TT: OR = 0.341, 95% CI = 0.168-0.693, *P* = 0.003; dominant model: OR = 0.384, 95% CI = 0.216-0.684, *P* = 0.001).

While compared with TT genotype of rs80177647, A allele carriers have increased EH risk (additive model: TA: OR = 2.119, 95% CI = 1.210-3.711, *P* = 0.009; dominant model: OR = 2.100, 95% CI = 1.226-3.596, *P* = 0.007). However, no significant association was observed after adjustment.

### 3.4. Association of PVT1 Polymorphisms and Risk for EH in China

Then, we aggregated and analyzed all the data from the two centers in southern and northern China. We found that the polymorphisms of the two SNPs of PVT1 were associated with the risk of EH. Logistic regression analysis showed that rs10956390 CT and TT genotypes were associated with decreased risk of EH (additive model: CT: OR = 0.596, 95% CI = 0.442-0.804, *P* = 0.001; TT: OR = 0.502, 95% CI = 0.350-0.718, *P* < 0.001; dominant model: OR = 0.564, 95% CI = 0.426-0.747, *P* < 0.001; recessive model: OR = 0.688, 95% CI = 0.506-0.936, *P* = 0.017, Table 5). And after adjustment, the association was still significant (additive model: CT: OR = 0.488, 95% CI = 0.326-0.729, *P* < 0.001; TT: OR = 0.460, 95% CI = 0.286-0.740, *P* = 0.001; dominant model: OR = 0.478, 95% CI = 0.328-0.698, *P* < 0.001).

Rs80177647 has similar results. The TA genotype and the dominant model showed stronger relations with higher EH risk (*P* < 0.001, OR = 1.993, 95% CI = 1.410-2.816; *P* < 0.001, OR = 1.986, 95% CI = 1.422-2.774, respectively). When adjusted for EH risk factors, including age, gender, BMI, smoking history, FBG, and dyslipidemia, the significant association between PVT1 rs80177647 was also observed (additive model: TA: OR = 1.768, 95% CI = 1.144-2.731, *P* = 010; dominant model: OR = 1.723, 95% CI = 1.128-2.631, *P* = 0.012).

### 3.5. Influence of PVT1 rs10956390 C>T and rs80177647 T>A Polymorphisms of Lipid Levels in Subjects

**Figure 1:**
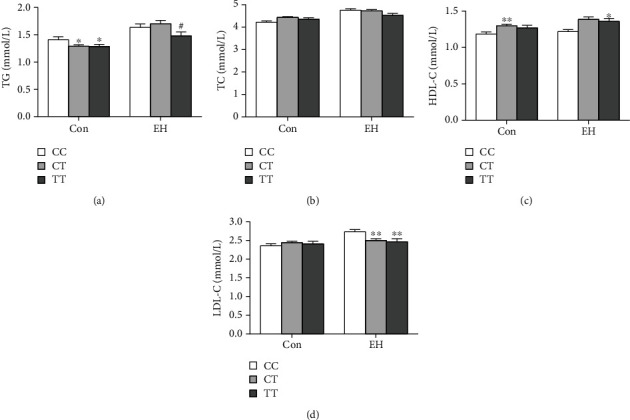
Influence of PVT1 rs10956390 polymorphism on lipid level of subjects: (a) TG; (b) TC; (c) HDL-C; (d) LDL-C. (Data are expressed as the mean ± SEM. Control: CC, *n* = 108, CT, *n* = 220, TT, *n* = 111; EH: CC, *n* = 192, CT, *n* = 233, TT, *n* = 99. ^∗^*P* < 0.05, ^∗∗^*P* < 0.01, as compared with CC genotype in interclass; ^#^*P* < 0.05, as compared with CT genotype in interclass).

**Figure 2:**
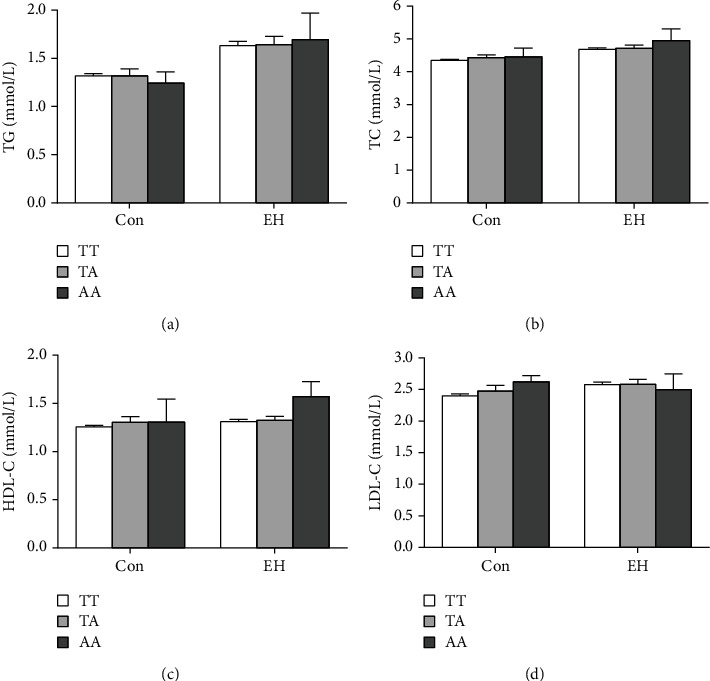
Influence of PVT1 rs80177647 polymorphism on lipid level of subjects: (a) TG; (b) TC; (c) HDL-C; (d) LDL-C. (Data are expressed as the mean ± SEM. Control: TT, *n* = 377, TA, *n* = 57, AA, *n* = 5; EH: TT, *n* = 395, TA, *n* = 119, AA, *n* = 10.).

Lipid levels, such as triglyceride, total cholesterol, high-density lipoprotein, and low-density lipoprotein were determined in 963 EH patients and normal controls. Moreover, the effects of rs10956390 and rs80177647 of PVT1 genotypes on lipid levels were analyzed. In controls, TG levels in CT and TT genotypes were significantly lower than those in subjects for rs10956390 CC genotype (^∗^*P* < 0.05, Figure 1(a)). And in EH patients, TG levels in TT genotype were lower than CT genotype (#*P* < 0.05, Figure 1(a)). More interestingly, we found that HDL-C levels in CT and TT genotypes were higher than CC genotype in control and EH patients, respectively (^∗^*P* < 0.05, ^∗∗^*P* < 0.01, Figure 1(c)). What is more, the levels of LDL-C were lower in CT and TT genotypes in EH patients (^∗∗^*P* < 0.01, compared with CC genotype, Figure 1(c)). However, by comparing the correlation between rs80177647 and lipid level in different subjects, we did not find significant differences between lipid levels and genotypes (Figure 2).

### 3.6. Association of PVT1 rs10956390 and rs80177647 Polymorphisms with PVT1 Expression

**Figure 3:**
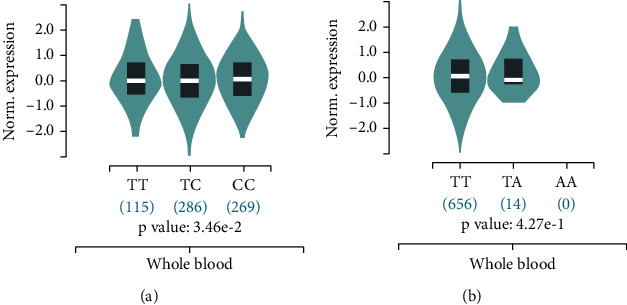
Effect of PVT1 mRNA expression in whole blood from healthy normal donors: (a) rs10956390; (b) rs80177647.

The Genotype-Tissue Expression (GTEx) project is one of the most widely used resources for studying the relationship between genetic variation and gene expression. This dataset contains genotype data from 838 postmortem donors and 17,382 RNA-seq samples across 54 tissue sites and 2 cell lines. We accessed the database to determine whether rs10956390 and rs80177647 polymorphisms were associated with PVT1 expression in multitissue through the GTEx eQTL Dashboard (https://www.gtexportal.org/home/eqtlDashboardPage). As shown in Figure 3(a), rs10956390 T allele was associated with decreased PVT1 mRNA in whole blood from 670 healthy donors (the relative expression median: TT: -0.04297, TC: -0.05181, CC: 0.02802, *P* = 0.0346). But there was no significant difference between rs80177647 polymorphism and PVT1 mRNA expression (Figure 3(b), *P* = 0.427).

## 4. Discussion

This study reveals, for the first time, there is an association between rs80177647 and rs10956390 gene polymorphisms of PVT1 and the risk of essential hypertension in two populations in southern and northern China. Our results demonstrated that EH risk was significantly decreased in carriers of T allele of rs10956390 polymorphism than those with the CC genotype. In addition, we also found that EH risk was significantly increased in carriers of A allele of rs80177647 polymorphism than those with TT.

The majority of studies in the past have focused on the relationship between PVT1 and the occurrence and development of various cancers. It is reported that PVT1 is typically upregulated in many types of cancer samples [20]. Some of those have found that PVT1 was involved in the regulation of angiogenesis in tumor tissues. Ma et al. showed that PVT1 overexpression promoted the proliferation, migration, and angiogenesis of glioma vascular endothelial cells [21]. However, a few recent studies have implied that PVT1 was involved in cardiovascular diseases. For instance, PVT1 acted as a sponge for miR-128-3p to facilitate Sp1 expression, resulting in activating the TGF-*β*1/Smad signaling pathway and regulating the development of atrial fibrosis [22]. Zhang et al. also found that PVT1 expression was significantly upregulated in abdominal aortic tissues from AAA patients, and knockdown PVT1 in AngII-induced AAA murine model suppresses VSMC apoptosis, ECM disruption, and serum proinflammatory cytokine level [23]. Strikingly, Quan et al. revealed that PVT1 was proved to be an independent risk factor for coronary atherosclerosis disease [24]. Thus, all those researches indicated the role of PVT1 involved in diseases is related to angiogenesis and endothelial dysfunction.

Previous studies in the association between PVT1 polymorphisms and diseases were only reported in the field of cancer and kidney disease. Our present data found two new SNP loci, rs10956390 and rs80177647, which polymorphisms were related to the risk of EH in Chinese population. However, the underlying mechanism is still unclear. lncRNAs play crucial roles in many human diseases; their structure and subcellular localization determine their functions [25]. A large amount of evidence has been demonstrated that the causality relationship between higher grade disequilibrium in endothelial homeostasis and diabetes or hypertension. The risky factors from vascular endothelial cell including reduced plasma nitric oxide level, suppressed L-arginine/endothelial nitric oxide synthase (eNOS) pathway, generation of reactive oxygen species (ROS), and enhanced leukocyte adhesion to the vascular wall may cooperatively contribute to the pathogenesis and progress of EH [26]. In our study, there were significant differences in TG, LDL-C, and HDL-C levels among rs10956390. Similarly, Wang et al. revealed that APOE-E3 homozygote and APOE-E4 allele were related to elevated triglycerides level; in addition, APOE-E2 allele was correlated with increased serum UA level in patients with hypertension or coronary heart disease [27]. Pan et al. found that ACE2 rs4646188 and rs879922 were associated with increased LDL-C level, while rs2106809 and rs4646188 were associated with hypertriglyceridemia [28]. The evidence we mentioned above demonstrates that different alleles in one gene may affect blood lipids differently. Most importantly, numerous studies have been revealed that there is a positive correlation between dyslipidemia and hypertension [29, 30]. A newly published study found that PVT1 was upregulated in the adipose tissue of obese mice and accelerated lipid accumulation by increasing the expression of peroxisome proliferator-activated receptor *γ*, CCAAT/enhancer-binding protein *α*, and adipocyte protein 2. In addition, PVT1 promoted fatty acid synthesis and inhibited fatty acid oxidation [17]. Based on all those studies and our data, we speculate that PVT1 may affect the risk of EH by regulating human blood lipid levels.

Several limitations of this study should be considered. Firstly, a limited number of samples were chosen in this study. Secondly, only the association between the PVT1 polymorphisms of the two loci, the risks of EH and plasma lipid levels have been investigated. However, the precise mechanism of how PVT1 influences lipid level in vivo or in vitro has not been studied. Further mechanism studies and larger population-based prospective studies are required.

## 5. Conclusion

In summary, our study demonstrates firstly that the PVT1 rs10956390 and rs80177647 polymorphisms are associated with the risk of EH in Chinese population. We speculate that the rs10956390 polymorphism is responsible for dyslipidemia. PVT1 is a potential biomarker and target for therapeutic strategies in EH. Further investigations in larger cohorts are needed to confirm our findings. More functional experiments are also required to illuminate the function role of rs10956390 and rs80177647 polymorphisms.

## Figures and Tables

**Table 1:** General characteristics of the EH case and control population.

Characteristics	The south of China			The north of China			Total		
	Control	EH	*P*	Control	EH	*P*	Control	EH	*P*
Gender	294	261		145	263		439	524	
Male (%)	173 (46.6%)	157 (60.2%)		75 (51.7%)	130 (49.4%)		248 (56.5%)	287 (54.8%)	
Female (%)	121 (53.4%)	104 (39.8%)	0.754	70 (48.3%)	133 (50.6%)	0.658	191 (43.5%)	237 (45.2%)	0.593
Age (years)	52 ± 8	54 ± 8.0	0.098	53 ± 9	53 ± 8	0.844	53 ± 8	53 ± 8	0.223
BMI (kg/m^2^)	22.20 ± 1.63	24.76 ± 2.71	< 0.001	22.64 ± 1.84	24.94 ± 3.34	< 0.001	22.35 ± 1.71	24.85 ± 3.04	< 0.001
SBP (mmHg)	120 ± 12	136 ± 20	< 0.001	122 ± 10	138 ± 19	< 0.001	120 ± 11	137 ± 19	< 0.001
DBP (mmHg)	76 ± 8	85 ± 13	< 0.001	75 ± 9	80 ± 13	< 0.001	76 ± 8	83 ± 13	< 0.001
Smoking history (%)	38 (12.9%)	63 (24.1%)	0.001	20 (13.8%)	80 (30.4%)	< 0.001	58 (13.2%)	143 (27.3%)	< 0.001
FBG, mmoL/L	4.54 ± 0.48	5.4 ± 1.17	<0.001	4.75 ± 1.49	5.5 ± 0.87	<0.001	4.61 ± 0.95	5.45 ± 1.03	<0.001
TG, mmol/L	1.30 ± 0.46	1.86 ± 1.99	<0.001	1.36 ± 0.43	1.41 ± 0.72	0.412	1.32 ± 0.45	1.64 ± 0.89	<0.001
TC, mmol/L	4.42 ± 0.62	4.83 ± 0.92	<0.001	4.22 ± 0.63	4.57 ± 0.97	<0.001	4.36 ± 0.63	4.70 ± 0.95	<0.001
HDL-C, mmol/L	1.27 ± 0.35	1.28 ± 0.41	0.741	1.26 ± 0.33	1.36 ± 0.52	0.013	1.26 ± 0.34	1.32 ± 0.47	0.032
LDL-C, mmol/L	2.52 ± 0.62	2.74 ± 0.81	<0.001	2.19 ± 0.52	2.41 ± 0.75	<0.001	2.41 ± 0.61	2.58 ± 0.80	<0.001

EH: essential hypertension; BMI: body mass index; SBP: systolic blood pressure; DBP: diastolic blood pressure; FBG: fasting blood glucose; TC: total cholesterol; TG: triglyceride; HDL-C: high-density lipoprotein cholesterol; LDL-C: low-density lipoprotein cholesterol.

**Table 2:** Test results of Hardy-Weinberg Equilibrium among all PVT1 gene sites.

Site	South of China				North of China			
	Control		EH		Control		EH	
	*χ* ^2^	*P*	*χ* ^2^	*P*	*χ* ^2^	*P*	*χ* ^2^	*P*
rs10956390	0.06	0.81	0.78	0.38	0.05	0.83	3.09	0.08
rs80177647	1.30	0.25	0.001	0.96	1.53	0.22	0.11	0.74

**Table 3:** Association between lncRNA PVT1 polymorphisms and EH risk in the south of China.

Models	Genotypes	Control, *n* = 294 (%)	EH, *n* = 261 (%)	Unadjusted OR (95% CI)	*P* value	^∗^Adjusted OR (95% CI)	^∗^Adjusted *P* value
rs10956390							
Additive	CC	75 (25.5)	92 (35.2)	1.00 (reference)		1.00 (reference)	
	CT	149 (50.7)	120 (46.0)	0.657 (0.445-0.968)	0.034	0.610 (0.340-1.094)	0.097
	TT	70 (23.8)	49 (18.8)	0.571 (0.355-0.918)	0.021	0.657 (0.319-1.354)	0.254
Dominant	CC	75 (25.5)	92 (35.2)	1.00 (reference)		1.00 (reference)	
	CT/TT	119 (74.5)	169 (64.8)	0.629 (0.437-0.906)	0.013	0.623 (0.357-1.085)	0.094
Recessive	CC/CT	224 (76.2)	212 (81.2)	1.00 (reference)		1.00 (reference)	
	TT	70 (23.8)	49 (18.8)	0.740 (0.491-1.115)	0.150	0.911 (0.494-1.680)	0.766

rs80177647							
Additive	TT	253 (86.1)	201 (77.0)	1.00 (reference)		1.00 (reference)	
	TA	38 (12.9)	56 (21.5)	1.855 (1.181-2.914)	0.007	1.950 (1.023-3.718)	0.042
	AA	3 (1.0)	4 (1.5)	1.678 (0.371-7.585)	0.501	2.063 (0.183-23.305)	0.558
Dominant	TT	253 (86.1)	201 (77.0)	1.00 (reference)		1.00 (reference)	
	TA/AA	41 (13.9)	60 (23.0)	1.842 (1.188-2.855)	0.006	1.956 (1.041-3.676)	0.037
Recessive	TT/TA	291 (99.0)	257 (98.5)	1.00 (reference)		1.00 (reference)	
	AA	3 (1.0)	4 (1.5)	1.510 (0.335-6.809)	0.592	1.843 (0.163-20.785)	0.621

OR: odd ratio; CI: confidence interval; EH: essential hypertension. ^∗^Adjusted for age, gender, BMI, smoking history, FBG, and dyslipidemia.

**Table 4:** Association between lncRNA PVT1 polymorphisms and EH risk in the north of China.

Models	Genotypes	Control, *n* = 145 (%)	EH, *n* = 263 (%)	Unadjusted OR (95% CI)	*P* value	^∗^Adjusted OR (95% CI)	^∗^Adjusted *P* value
rs10956390							
Additive	CC	33 (22.8)	100 (38.0)	1.00 (reference)		1.00 (reference)	
	CT	71 (48.3)	113 (43.0)	0.525 (0.321-0.860)	0.010	0.409 (0.221-0.757)	0.004
	TT	41 (28.3)	50 (19.0)	0.402 (0.228-0.712)	0.002	0.341 (0.168-0.693)	0.003
Dominant	CC	33 (22.8)	100 (38.0)	1.00 (reference)		1.00 (reference)	
	CT/TT	112 (77.2)	163 (62.0)	0.480 (0.303-0.762)	0.002	0.384 (0.216-0.684)	0.001
Recessive	CC/CT	104 (71.7)	223 (81.0)	1.00 (reference)		1.00 (reference)	
	TT	41 (28.3)	50 (19.0)	0.595 (0.370-0.957)	0.032	0.598 (0.333-1.072)	0.084

rs80177647							
Additive	TT	124 (85.5)	194 (73.8)	1.00 (reference)		1.00 (reference)	
	TA	19 (13.1)	63 (24.0)	2.119 (1.210-3.711)	0.009	1.535 (0.802-2.938)	0.196
	AA	2 (1.4)	6 (2.3)	1.918 (0.381-9.652)	0.430	0.734 (0.109-4.944)	0.751
Dominant	TT	124 (85.5)	194 (73.8)	1.00 (reference)		1.00 (reference)	
	TA/AA	21 (14.5)	69 (26.3)	2.100 (1.226-3.596)	0.007	1.449 (0.775-2.710)	0.246
Recessive	TT/TA	143 (98.6)	257 (97.7)	1.00 (reference)		1.00 (reference)	
	AA	2 (1.4)	6 (2.3)	1.669 (0.333-8.379)	0.534	0.664 (0.098-4.483)	0.674

OR: odd ratio; CI: confidence interval; EH: essential hypertension. ^∗^Adjusted for age, gender, BMI, smoking history, FBG, and dyslipidemia.

**Table 5:** Association between lncRNA PVT1 polymorphisms and EH risk in China.

Models	Genotypes	Control, *n* = 439 (%)	EH, *n* = 524 (%)	Unadjusted OR (95% CI)	*P* value	^∗^Adjusted OR (95% CI)	^∗^Adjusted *P* value
rs10956390							
Additive	CC	108 (24.6)	192 (36.6)	1.00 (reference)		1.00 (reference)	
	CT	220 (50.1)	233 (44.5)	0.596 (0.442-0.804)	0.001	0.488 (0.326-0.729)	<0.001
	TT	111 (25.3)	99 (18.9)	0.502 (0.350-0.718)	<0.001	0.460 (0.286-0.740)	0.001
Dominant	CC	108 (24.6)	192 (36.6)	1.00 (reference)		1.00 (reference)	
	CT/TT	331 (75.4)	332 (63.4)	0.564 (0.426-0.747)	<0.001	0.478 (0.328-0.698)	<0.001
Recessive	CC/CT	328 (74.7)	425 (81.1)	1.00 (reference)		1.00 (reference)	
	TT	111 (25.3)	99 (18.9)	0.688 (0.506-0.936)	0.017	0.721 (0.483-1.077)	0.110

rs80177647							
Additive	TT	377 (85.9)	395 (75.4)	1.00 (reference)		1.00 (reference)	
	TA	57 (13.0)	119 (22.7)	1.993 (1.410-2.816)	<0.001	1.768 (1.144-2.731)	0.010
	AA	5 (1.1)	10 (1.9)	1.909 (0.646-5.636)	0.242	1.170 (0.250-5.40)	0.842
Dominant	TT	377 (85.9)	395 (75.4)	1.00 (reference)		1.00 (reference)	
	TA/AA	62 (14.1)	129 (24.6)	1.986 (1.422-2.774)	<0.001	1.723 (1.128-2.631)	0.012
Recessive	TT/TA	434 (98.9)	514 (98.1)	1.00 (reference)		1.00 (reference)	
	AA	5 (1.1)	10 (1.9)	1.689 (0.573-4.978)	0.342	1.045 (0.221-4.927)	0.956

OR: odd ratio; CI: confidence interval; EH: essential hypertension. ^∗^Adjusted for age, gender, BMI, smoking history, FBG, and dyslipidemia.

## Data Availability

The research data used to support the findings of this study are available from the corresponding author upon request.
